# Characteristic of acute and overuse injuries among junior, senior and master rowers

**DOI:** 10.1186/2052-1847-7-S1-O14

**Published:** 2015-08-11

**Authors:** Tomislav Smoljanovic, Ivan Bojanic, Jo A Hannafin, Darko Hren, Oliver Terborg, Ivan Bohacek, Henning Bay Nielsen

**Affiliations:** 1 Department of Orthopaedic Surgery, University Hospital Center Zagreb, School of Medicine, Zagreb University, Zagreb, Croatia; 2 Sports Medicine and Shoulder Service, Hospital for Special Surgery, Weill Medical College of Cornell University New York, New York, NY, USA; 3 Chair for Psychology, Faculty of Humanities and Social Sciences, University of Split, Split, Croatia; 4 Lt Colonel (Medical Corps), Forces Policy, Federal Ministry of Defence, Berlin, Germany; 5 Department of Anesthesia, Rigshospitalet, University of Copenhagen, Copenhagen, Denmark

**Keywords:** Rowing, acute injury, overuse injury, junior, senior, master

## Background

There was no comprehensive analysis of injuries among international elite-level rowers of different age categories. Objective of this study was to define the most frequent acute (traumatic) and chronic (overuse) musculoskeletal injuries in international elite-level junior, senior and master rowers.

## Materials and methods

1775 elite level rowers from 70 different countries participating at the 2007 Junior, Senior and Master World Rowing Championships were included in a survey on rowing-specific 4-page questionnaire related to potential injuries and data were compared to data obtained from previous seasons. The questionnaire was based on general data, rowing discipline, training program data and potential injuries according to an anatomical localization.

## Results

Among the 1775 rowers, 812 reported a total of 1343 injuries with 935 injuries related to a chronic or overuse effect on the musculoskeletal system. Low back pain was the most frequent complaint and a high incidence of chronic injuries was associated with increased average number of rowing sessions in both junior and senior rowers. Acute injuries (n= 398) were less frequent compared to chronic injuries and the majority were acquired during on-water rowing specific training while non-rowing specific injuries were provoked by football, basketball and volleyball activities. Indoor rowing training such as gym and weightlifting in addition to rowing ergometer training were associated to less frequent injuries. The risk of injury was calculated to 1.75 and 2.25 injuries/1000 training sessions/rower and injured junior rowers had rowed for a shorter time compared to the rowers who did not report injuries. Senior open-weight rowers who have sustained chronic injuries have achieved significantly better final ranking at 2007 Senior World Rowing Championships, compared to the same group of rowers who did not sustain any injury.

## Conclusions

The level of reported injuries reported in elite rowers is relatively high but considering the amount of training hours rowing is a low risk sport. Among all injuries in elite-level rowers, the predominant complain is low back pain.

